# Supplementary material for: Bioluminescent Zebrafish Transplantation Model for Drug Discovery
Source: Front Pharmacol. 2022 Apr 27;13:893655. doi: 10.3389/fphar.2022.893655 (PMC9086674; doi:10.3389/fphar.2022.893655)
Supplement: Supplementary file 1 [file DataSheet1.pdf]

SUPPLEMENT

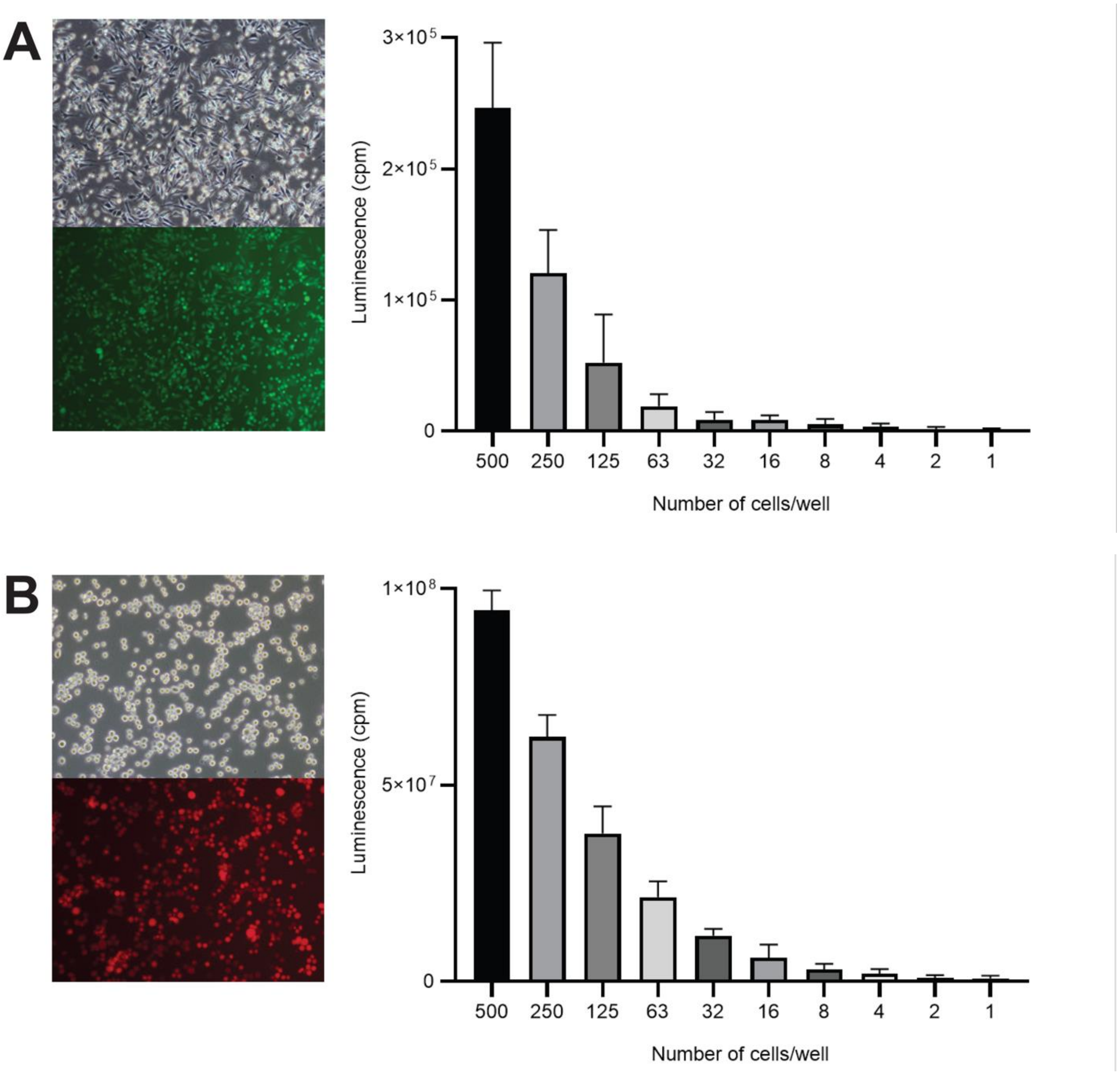

Figure S1. Cancer cells expressing a double reporter system can be tracked by fluorescence and luminescence. (A) ZMEL1-*EGFP-NLuc* cells in culture, on top brightfield, on bottom green cells expressing *EGFP*. The bar graph represents the amount of luminescence measured in cells according to x axis *in vitro*. (B) K562-*mCherry-NLuc* cells in culture, on top brightfield, on bottom red cells expressing *mCherry*. The bar graph represents the amount of luminescence measured in cells according to x axis *in vitro*. Images of cultures were acquired on Olympus IX70 microscope.

Table S1. Library of kinase inhibitors used in the screen with their *in vivo* and *in vitro* potency and predicted targets

			Potent *in vivo*		*in vitro* [µM] ZMEL1	*in vitro* [µM] K562		
**Inhibitor Name**	**SID**	**Toxicity *in vivo***	**ZMEL1**	**K562**	**IC50**	**IC50**	**Link to P&D**	**Predicted targets**
**Abemaciclib**	107544	N	Y	N	18.103	2.955	Abemaciclib	CDK4
**Afatinib**	40853	Y	ND	ND	ND	ND	Afatinib	EGFR, ERBB4
**Alectinib**	107806	N	N	N	17.197	too high	Alectinib	ALK, EML4
**Alisertib**	107564	Y	ND	ND	ND	ND	Alisertib	AURKA
**Alpelisib**	107801	N	N	N	3.690	30.541	Alpelisib	PIK3CA
**AMG-900**	106690	Y*	Y*	ND	0.003	too high	AMG-900	AURKA, AURKC
**AP26113**	107605	N	N	N	0.364	2.070	AP26113	ALK
**AT-9283**	106862	N	N	N	0.308	0.722	AT-9283	CDK10
**AZ 191**	107912	N	N	N	7.168	12.837	AZ 191	DYRK1B
**AZ20**	106930	N	Y	N	5.499	1.858	AZ20	ATR
**AZD-1208**	107740	N	N	N	32.534	8.275	AZD-1208	PIM1
**AZD-5438**	107724	N	N	N	2.792	2.379	AZD-5438	CDK2
**AZD-7762**	105064	Y*	Y*	Y*	0.283	0.202	AZD-7762	SIK2, CHEK1
**Bafetinib**	107621	N	Y	N	11.720	0.011	Lyn-IN-1	ABL, LYN
**Barasertib**	107791	N	Y	N	31.407	too high	Barasertib	AURKC, AURKB
**Baricitinib**	107751	N	N	N	too high	34.735	Baricitinib	JAK1, JAK2
**BAY-474**	108059	N	N	N	too high	too high	BAY-474	MET
**BAY-826**	108056	N	N	Y	too high	0.458	BAY-826	DDR1, TEK/TIE2
**Berzosertib**	107683	N	N	N	3.704	0.819	Berzosertib	ATR
**BI 1002494**	105963	N	N	N	too high	8.570	BI 1002494	SYK
**BI 2536**	107634	N	N	N	2.144	0.047	BI 2536	PLK1
**BI 831266**	105979	N	N	N	81.979	3.410	BI 831266	AURK8
**BI-1347**	105966	Y	ND	ND	ND	ND	BI-1347	CDK8
**BI-1347**	110888	Y	ND	ND	ND	ND	BI-1347	CDK8
**BI-2536**	105969	Y	ND	ND	ND	ND	BI 2536	PLK1

**BI-D1870**	106683	Y	ND	ND	ND	ND	BI-D1870	RPS6KA6, RPS6KA2
**BIX02188**	107654	N	N	Y	21.153	26.194	BIX02188	MAP2K5/MEK5
**BLU9931**	87330	Y	ND	ND	ND	ND	BLU9931	FGFR4
**BMS-345541**	106698	N	N	N	21.612	6.043	BMS-345541	IKBKB
**BMS-509744**	105913	N	N	N	4.773	5.390	BMS-509744	ITK
**BMS-536924**	106888	N	N	N	0.391	5.507	BMS-536924	IGF1R
**BMS-777607**	107730	Y	ND	ND	ND	ND	BMS-777607	TYRO3, AXL
**BMS-911543**	106734	N	N	N	too high	too high	BMS-911543	JAK2
**Bosutinib**	105848	Y*	ND	Y*	6.993	0.029	Bosutinib	MAP4K5, TXK
**B-Raf inhibitor 1**	107338	Y	ND	ND	ND	ND	B-Raf inhibitor 1	BRAF
**Brivanib**	106793	Y	ND	ND	ND	ND	Brivanib	VEGFR2
**Cabozantinib**	106804	Y	ND	ND	ND	ND	Cabozantinib	KDR
**Canertinib**	107718	N	N	N	73.367	36.830	Canertinib	EGFR
**Capmatinib**	107800	Y	ND	ND	ND	ND	Capmatinib	MET
**Cediranib**	106864	Y	ND	ND	ND	ND	Cediranib	KIT, KDR
**Ceritinib**	107670	Y	ND	ND	ND	ND	Ceritinib	ALK
**CGI1746**	107745	N	N	N	too high	14.321	CGI1746	BTK
**Cobimetinib**	106849	Y*	Y*	ND	0.012	0.639	Cobimetinib	MAP2K1
**CP-673451**	106808	N	N	N	6.232	too high	CP-673451	PDGFRB
**CP-724714**	106765	N	Y	N	18.423	33.535	CP-724714	ERBB2
**CRT 0066101**	107005	N	Y	N	1.647	4.870	CRT 0066101	PRKD1
**Dabrafenib**	107825	Positive control	Positive control	ND	0.067	ND	Dabrafenib	ARAF, BRAF
**Dactolisib**	107587	Y	ND	ND	ND	ND	Dactolisib	ATM, PIK3CA
**DDR-IN-1**	106879	N	N	Y	6.788	0.404	DDR1-IN-1	DDR1
**Defactinib**	108072	N	N	N	23.247	25.374	Defactinib	PTK2
**Doramapimod**	106928	N	Y	N	18.303	32.656	Doramapimod	MAPK12, MAPK14
**Ensartinib**	105141	N	N	N	4.945	0.667	Ensartinib	MAP4K2, ALK, MET
**ERKi**	108026	N	N	N	0.186	too high	ERKi	MAPK3, MAPK1
**Erlotinib**	107324	Y	ND	ND	ND	ND	Erlotinib	MAP3K19, EGFR

**Fasudil**	107603	N	N	N	70.722	too high	Fasudil	PKN3
**Filgotinib**	40830	N	N	N	11.512	10.763	Filgotinib	JAK1
**FM-381**	105530	Y	ND	ND	ND	ND	FM-381	JAK3
**Fruquintinib**	107787	Y	ND	ND	ND	ND	Fruquintinib	FLT4
**G-749**	40828	Y*	Y*	ND	0.802	too high	G-749	FLT3
**Galunisertib**	40846	N	N	N	too high	too high	Galunisertib	ACVR1B, RIPK3
**GDC-0941**	40811	Y	ND	ND	ND	ND	GDC-0941	PIK3CA, PIK3CD
**Gedatolisib**	107815	N	Y	N	0.114	0.163	Gedatolisib	PIK3C2B, PIK3CA
**Gilteritinib**	106738	N	Y	N	0.972	too high	Gilteritinib	FLT3, ULK3, LTK
**GNE-3511**	106713	Y	ND	ND	ND	ND	GNE-3511	MAP3K12, MAP3K13
**GNE-7915**	106752	N	N	N	51.827	too high	GNE-7915	LRRK2, TTK
**GNF-5**	107689	N	N	Y	35.294	0.696	GNF-5	ABL1
**GNF-5837**	106896	Y	ND	ND	ND	ND	GNF5837	NTRK2, NTRK3
**Go 6976**	41025	N	Y	N	0.050	0.568	Go 6976	FLT3, PRKCG (PKC), JAK2
**GSK1838705**	107772	N	N	N	0.315	9.047	GSK1838705	ALK
**GSK2334470**	107762	N	N	N	1.623	20.138	GSK2334470	PDPK1
**GSK2606414**	107769	N	N	N	55.333	15.157	GSK2606414	EIF2AK3
**GSK269962A**	106924	Y	ND	ND	ND	ND	GSK269962A	ROCK1
**GSK429286A**	107783	N	N	N	too high	32.242	GSK429286A	ROCK1
**GSK461364**	106747	N	N	N	6.701	0.012	GSK461364	PIP5K1A, PLK1
**GSK579289A**	105069	N	Y	N	5.721	0.039	GSK579289A	PLK1
**GSK583**	106932	N	N	N	33.112	44.291	GSK583	RIPK2
**GSK650394**	107779	Y	ND	ND	ND	ND	GSK650394	CAMKK2, SGK1
**GTPL8068**	107687	N	N	N	4.378	0.344	GTPL8068	LCK
**GTPL8215**	106682	N	N	N	35.016	2.100	GTPL8215	CHEK1
**GW843682X**	106847	N	N	N	too high	0.636	GW843682X	PLK1
**CHIR-98014**	107767	N	N	N	1.986	too high	CHIR-98014	GSK3B
**CHIR-99021**	41029	N	N	N	8.897	5.360	CHIR-99021	GSK3B, GSK3B
**Ibrutinib**	106982	Y	ND	ND	ND	ND	Ibrutinib	BTK

**Imatinib mesylate**	39808	Positive control	ND	Positive control	ND	0.147	Imatinib	ABL1, BCR, CF1R
**Infigratinib**	107810	N	N	N	7.822	too high	Infigratinib	FGFR4, FGFR1
**Ipatasertib**	40848	N	N	Y	0.193	13.243	Ipatasertib	AKT1, AKT3
**JNK-IN-8**	40858	N	N	N	7.882	6.786	JNK-IN-8	MAPK10
**KU-0060648**	105058	N	Y	N	21.852	3.840	KU 0060648	PIK3CD, PRKDC
**KU-60019**	107707	N	N	N	too high	15.813	KU 60019	ATM
**Lenvatinib**	107380	Y	ND	ND	ND	ND	Lenvatinib	RIPK2, RET
**Linsitinib**	40342	Y*	Y*	ND	0.093	16.364	Linsitinib	IGF1R, INSR
**Losmapimod**	40831	N	N	N	too high	too high	Losmapimod	MAPK14, STK24
**LRRK2-IN-1**	107590	N	N	N	24.104	1.395	LRRK2-IN-1	LRRK2
**LY-2090314**	106764	Y	ND	ND	ND	ND	LY-2090314	GSK3B, GSK3A
**LY-2109761**	106949	N	N	N	too high	46.877	LY2109761	TGFBR1
**LY-3009120**	106727	Y*	Y*	ND	0.132	0.441	LY3009120	KRAS, BRAF
**Maisitinib**	107768	N	N	N	9.395	1.273	MASITINIB	SLC25A6, KIT, CSF1R
**Midostaurin**	105033	Y	ND	ND	ND	ND	Midostaurin	TBK1, FLT3
**MK-1775**	107545	N	N	N	84.078	0.858	MK-1775	WEE1
**MK-5108**	106893	Y*	ND	Y*	too high	0.285	MK-5108	AURKA
**ML-120B**	106706	N	N	N	too high	too high	ML-120B	IKBKB
**Momelotinib**	107657	N	N	N	21.139	4.178	Momelotinib	JAK1
**MPS1-IN-1**	106911	N	N	N	17.018	7.053	MPS1-IN-1	TTK
**MRL-SYKi**	108022	N	N	Y	too high	too high	MRL-SYKi	SYK, ZAP70
**MRT67307**	106836	N	N	N	51.739	9.005	MRT67307	MARK1, TBK1
**Neflamapimod**	107907	N	N	N	25.797	5.740	Neflamapimod	MAPK14
**NG-25**	105042	Y*	ND	Y*	4.161	0.003	NG-25	MAP4K2, LYN, TAK1
**Nilotinib**	107502	Y	ND	ND	ND	ND	Nilotinib	DDR2, ABL1
**NVP-AEW541**	40849	Y*	Y*	ND	0.651	4.262	NVP-AEW541	IGF1R, ATR
**NVS-PAK1-1**	89266	Y	ND	ND	ND	ND	NVS-PAK-1	PAK1
**Omipalisib**	107764	Y	ND	ND	ND	ND	Omipalisib	PIK3R1
**Palbociclib**	42207	N	N	N	5.692	7.070	Palbociclib	CDK4

**Pazopanib**	107581	N	Y	N	24.476	7.687	Pazopanib	PDGFRA
**PD-023177**	107752	N	N	N	6.571	2.879	PD-023177	ND
**PD-0325901**	107631	Y	ND	ND	ND	ND	PD-0325901	MAP2K1
**PD-0325901**	41030	Y	ND	ND	ND	ND	PD-0325901	MAP2K2, MAP2K1
**PD-153035**	107701	Y	ND	ND	ND	ND	PD-153035	EGFR
**Pelitinib**	107614	Y	ND	ND	ND	ND	Pelitinib	MAP4K5, EGFR
**PF-00477736**	107921	N	N	N	11.007	0.375	PF-00477736	CHEK1
**PF-03758309**	106740	N	N	N	0.072	0.003	PF-03758309	PAK1, PAK4
**PF-3644022**	105843	N	N	N	1.818	0.084	PF-3644022	MAPKAPK2
**PF-4708671**	107697	N	N	N	45.964	34.095	PF-4708671	RPS6KB1
**PF-4800567**	105093	N	N	N	too high	too high	PF-4800567	CSNK1E
**PHA-665752**	106845	N	N	N	1.593	9.168	PHA-665752	SRPK1, MET
**PHA-793887**	107573	N	Y	N	5.687	0.177	PHA-793887	CDK5R1, multiple CDKs
**PI-103**	106946	Y	ND	ND	ND	ND	PI-103	PRKDC, PIK3CA
**Pictilisib**	40859	N	N	Y	0.944	4.598	Pictilisib	PIK3CA, PIK3CD
**PKC 412**	107857	Y	ND	ND	ND	ND	PKC 412	PKN1, FLT3
**PLX-4720**	106741	N	Y	N	1.615	31.312	PLX-4720	MAP2K5, KDR
**Ponatinib**	107549	Y	ND	ND	ND	ND	Ponatinib	ABL1, LYN
**PP-121**	107725	Y*	Y*	ND	0.269	0.581	PP-121	HCK, PDGFRA
**PP2**	12583	Y	ND	ND	ND	ND	PP2	LCK
**Prexasertib**	105142	N	N	N	7.743	0.021	Prexasertib	CHEK1
**Quizartinib**	40851	Y	ND	ND	ND	ND	Quizartinib	FLT3, KIT
**Ravoxertinib**	106797	N	N	N	too high	too high	Ravoxertinib	MAPK3, MAPK1
**RG-1530**	106742	Y	ND	ND	ND	ND	RG-1530	KDR, FLT1
**Ribociclib**	107874	N	Y	N	too high	too high	Ribociclib	CCND3, CDK4, CDK6
**RKI-1447**	107824	N	N	Y	19.428	28.665	RKI-1447	ROCK2, ROCK1
**RO-4584820**	105072	N	N	N	2.308	1.902	RO-4584820	CCND1, CDK4
**Roscovitine**	107736	N	Y	N	24.331	too high	Roscovitine	CDK5, CDK2
**Ruboxistaurin**	107906	Y	ND	ND	ND	ND	Ruboxistaurin	PRKCQ
**Ruxolitinib**	40843	N	N	Y	43.321	5.291	Ruxolitinib	JAK1, JAK2

**Sapitinib**	107918	Y	ND	ND	ND	ND	Sapitinib	EPHA1, EGFR
**SAR405**	106787	N	N	N	too high	26.246	SAR405	PIK3C3
**SB-202190**	106889	N	N	Y	too high	too high	SB 202190	NLK, MAPK14
**SB-203580**	40850	N	Y	Y	too high	too high	SB 203580	MAPK14 (p38a), NLK
**SB-431542**	41004	N	Y	N	1.976	too high	SB 431542	TGFBR1/2 (ALK5)
**SB-590885**	104721	N	Y	N	3.412	7.356	SB 590885	RAF1, BRAF
**SB-590885**	107735	N	N	N	44.678	too high	SB 590885	RAF1, BRAF
**SGI-1776**	107773	Y	ND	ND	ND	ND	SGI-1776	PRKAG2, PIM1
**SGX-523**	107570	N	N	N	too high	too high	SGX-523	MET
**SCH772984**	107639	N	N	N	0.059	0.250	SCH772984	MAPK1
**Silmitasertib**	107594	Y	ND	ND	ND	ND	Silmitasertib	CSNK2A1
**SL-327**	107733	N	Y	N	13.069	too high	SL-327	MAP2K2 (MEK2), MAPK1
**Sotrastaurin**	106826	N	N	N	19.253	too high	Sotrastaurin	PRKCD, PRKCQ
**Spebrutinib**	107748	N	N	Y	30.779	4.183	Spebrutinib	BTK
**STO-609**	107889	Y	ND	ND	ND	ND	STO-609	CAMKK2
**TAK-715**	106874	N	Y	N	56.997	14.126	TAK-715	MAPK14
**Tamatinib**	106941	N	N	N	21.129	11.322	Tamatinib	MAP3K10, RET
**Taselisib**	107694	N	N	N	0.127	24.600	Taselisib	PIK3CD
**TG100-115**	107523	N	N	N	too high	too high	TG100-115	PIK3CG
**TH257**	105531	N	N	N	57.065	too high	TH257	LIMK1, LIMK2
**THZ1**	107913	N	N	Y	6.970	0.064	THZ1	CDK7
**Tivozanib**	107684	Y	ND	ND	ND	ND	Tivozanib	PEBP1, KDR
**Tofacitinib**	107522	N	N	N	too high	too high	Tofacitinib	JAK2, JAK3
**Torin 1**	107636	Y	ND	ND	ND	ND	Torin 1	MTOR
**Torin 2**	107828	Y*	Y*	ND	0.054	0.055	Torin 2	MTOR, PRKDC
**Torkinib**	106971	N	Y	N	0.291	1.856	Torkinib	BMPR1B, MTOR
**Trametinib**	107744	Y	ND	ND	ND	ND	Trametinib	MAP2K1, MAP2K2
**Ulixertinib**	107040	Y*	Y*	ND	0.247	11.546	Ulixertinib	MAPK1 (ERK2)
**UNC2025**	106871	N	N	N	3.061	2.056	UNC2025	FLT3
**VE-821**	107753	N	N	N	too high	33.804	VE-821	ATR

**Vertex 11e**	107852	Y*	Y*	ND	0.115	5.202	VX-11e	MAPK1
**Volasertib**	107917	N	N	N	1.086	0.035	Volasertib	PLK1
**VS-4718**	107796	N	N	N	16.141	4.066	VS-4718	MAPK10, PTK2
**VX-702**	107583	N	Y	N	too high	5.632	VX-702	MAPKAPK2
**WZ4002**	106716	Y	ND	ND	ND	ND	WZ4002	EGFR, ERBB4
**WZ4003**	106788	N	N	N	8.210	8.013	WZ4003	NUAK1, NUAK2
**XMD8-92**	107555	Y	ND	ND	ND	ND	XMD8-92	MAPK7
**ZM-447439**	106696	N	N	Y	9.286	too high	ZM-447439	EGFR, AURKA, AURKB

* not at 1 uM	Potent	Potent
	Not significant	Not significant
	Not potent	Not potent
	Not determined	Not determined
	* at 1 µM	* at 1 µM

Table S2. Hit compounds from *in vivo* screen with the expression of their predicted main targets

		Inhibitor	Predicted main targets	Expression of main target [rlog]
ZMEL1	RAS & p38 MAPK pathways	Doramapimod	Mapk12a	10.3
			Mapk14a	10.9
			Mapk14b	9.8
		Gedatolisib	Pik3c2a	12.7
			Pik3c2b	8.9
			Pik3ca	9.9
		PLX-4720	Braf	9.0
			Map2k5	9.5
		SB-203580	Mapk14a	10.9
			Mapk14b	9.8
		SL-327	Map2k2a	12.1
			Map2k2b	11.0
			Mapk1	9.5
		TAK-715	Mapk14a	10.9
			Mapk14b	9.8
		Torkinib	Bmpr1ba	6.0
			Bmpr1bb	6.3
			Mtor	12.7
		Cobimetinib	Map2k1	9.7
		LY3009120	Braf	9.0
			Kras	9.9
		Torin 2	Mtor	12.7
			Prkdc	11.4
		Ulixertinib	Mapk1	9.5
	Cell cycle	Abemaciclib	Cdk4	10.7
		Barasertib	Aurkb	12.6
		GSK579289A	Plk1	12.1
		PHA-793887	Cdk2	10.7
			Cdk5	9.2
		Ribociclib	Ccnd3	5.4
			Cdk4	10.7
			Cdk6	11.3
		Roscovitine	Cdk2	10.7
			Cdk5	9.2
		AMG-900	Aurka	10.7
			Aurkb	12.6

		AZD-7762	Chek1	9.4
			Chek2	9.4
			Sik2a	4.8
			Sik2b	12.7
	RTKs	CP-724714	Erbb2	7.6
		Go 6976	Flt3	5.9
			Prkcaa	12.1
			Prkcab	7.7
		Pazopanib	Fgfr1a	8.7
			Fgfr1b	6.3
			Kdr	6.2
			Pdgfra	8.4
			Pdgfrb	9.3
		SB-431542	Tgfbr1a	7.8
			Tgfbr1b	8.5
		G-749	Flt3	5.9
		Linsitinib	Igf1ra	10.6
			Igf1rb	10.0
			Insra	11.0
			Insrb	10.4
		PP-121	Hck	8.9
			Kdr	6.2
			Mtor	12.7
			Pdgfra	8.4

Table continued on the next page

		Inhibitor	Predicted main targets	Expression of main target [rlog]
K562	RAS & p38 MAPK pathways	BIX02188	MAP2K5	8.5
		GNF-5	ABL1	11.5
			BCR	12.1
		Ipatasertib	AKT1	10.7
			AKT3	11.1
		Pictilisib	PIK3R4	9.4
		SB-202190	MAPK14	11.1
		SB-203580	MAPK14	11.1
		Bosutinib	MAP4K5	9.5
			TXK	5.6
		NG-25	LYN	10.4
			MAP3K7	10.3
			MAP4K2	8.1
	Cell cycle & migration	BAY-826	DDR1	7.9
			TEK	1.7
			TIE1	4.0
		DDR-IN-1	DDR1	7.9
		RKI-1447	ROCK1	10.7
			ROCK2	10.5
		Ruxolitinib	JAK1	11.2
			JAK2	10.5
			JAK3	3.4
		THZ1	CDK7	8.4
		ZM-447439	AURKA	9.8
			AURKB	9.3
		AZD-7762	CHEK1	10.8
			CHEK2	8.7
			SIK2	9.8
		MK-5108	AURKA	9.8
